# Management of work-related common mental disorders in general practice: a cross-sectional study

**DOI:** 10.1186/s12875-020-01203-z

**Published:** 2020-07-02

**Authors:** M. Rivière, Y. Toullic, P. Lerouge, T. Blanchon, A. Leroyer, L. Plancke, T. Prazuck, M. Melchior, N. Younès

**Affiliations:** 1 Sorbonne Université, INSERM, Institut Pierre Louis d’Épidémiologie et de Santé Publique, IPLESP, 75012 Paris, France; 2 Department of Infectious Diseases, Réseau Sentinelles, Centre Hospitalier Régional, d’Orléans 14 avenue de l’hôpital, 45000 Orléans, France; 3 University department of general practice, Université Lille 2, Lille, France; 4 Univ. Lille, CHU Lille, Institut Pasteur de Lille, EA 4483 - IMPECS - IMPact de l’Environnement Chimique sur la Santé humaine, F-59000 Lille, France; 5 Regional Federation of Research in Psychiatry and Mental Health Hauts-de-France, Lille, France; 6 Université de Versailles Saint-Quentin, Université Paris Saclay, CESP, Team DevPsy, 94807 Villejuif, France; 7 Centre Hospitalier de Versailles, Hospital Academic Unit of psychiatry for adults, Le Chesnay, France

**Keywords:** Common mental disorders, Work, Management, Referral, General practice

## Abstract

**Background:**

General practitioners (GPs) often manage individuals with work-related common mental disorders (CMD: depressive disorders, anxiety and alcohol abuse). However, little is known about the ways in which they proceed. The aim of this study is to analyze GPs’ management and patterns of referral to other health professionals of patients with work-related CMD and associated factors.

**Method:**

We used data from a cross-sectional study of 2027 working patients of 121 GPs in the Nord – Pas-de-Calais region in France (April – August 2014). Statistical analyses focused on patients with work-related CMD detected by the GP and examined the ways in which GPs managed these patients’ symptoms. Associations between patient, work, GP and contextual characteristics and GPs’ management were explored using modified Poisson regression models with robust variance.

**Results:**

Among the 533 patients with work-related CMD in the study, GPs provided psychosocial support to 88.0%, prescribed psychotropic treatment to 82.4% and put 50.7% on sick leave. Referral rates to mental health specialists and occupational physicians were respectively 39.8 and 26.1%. Several factors including patients’ characteristics (occupational and sociodemographic), GPs’ characteristics and environmental data were associated with the type of management used by the GP.

**Conclusion:**

Our study emphasizes the major and often lonesome role of the GP in the management of patients with work-related CMDs. Better knowledge of the way GPs manage those patients could help GPs in their practice, improve patients care and be a starting point to implement a more collaborative care approach.

## Background

Work-related mental disorders have been defined by WHO in 1985 as “multifactorial diseases which may frequently be work-related but also occurring among the general population. They may be partially caused by adverse working conditions, aggravated, accelerated or exacerbated by work-place exposures or they can impair work ability”. The definition is vague as adverse work conditions can be a cause and a consequence of this work related-disorder. Mental health disorders, including common mental disorders (CMD), ie depression, anxiety and substance use disorders represent the second leading cause of work-related diseases and the first cause of work-related sickness absence [1–3]. Work-related common mental disorders have been described in occupational medicine literature [2–6] and primary care [7–10], but patterns of health-care access for patients with work-related mental health disorders use in primary care is not well-known. In many cases the GP is not only the first but also the only health professional contacted [7, 11–15].

According to the WHO’s Service Organization Pyramid for an Optimal Mix of Services for Mental Health, every family should have a family doctor, with access to a range of specialist services, both in community and hospital settings. The position of the GP varies considerably across countries and health care systems, as well as depending on patients’ expectations and GPs’ task performance. In some countries such as the Netherlands, Spain and the UK, the GP is a gatekeeper to specialists and the treatment of patients with psycho-social problems is primarily GPs’ task. In others countries such as France, Belgium, Germany and Switzerland, patients can access a specialist directly and visiting the GP is optional [7, 11–15]. However, in France, visiting a GP is a frequent option because of availability and health insurance coverage, which in most cases is at best partial for mental health specialists.

The prevalence of mental disorders in primary care settings has been researched extensively in different countries. Over the years, the prevalence among adults has been documented to range between 10 and 60%; mainly depression (ranging from 5 to 20%), generalized anxiety disorder (4 to 15%), harmful alcohol use and dependence (5 to 15%), and somatization disorders (0.5 to 11%). In France, primary care is available and covered by the French National Health Insurance system and 2/3 GPs offer psychological support to patients who are depressed [16–18]. They also prescribe drug therapy: antidepressants, anxiolytics or hypnotics [19–21] and psychological treatment [22]. The NICE guidelines recommend that patients with mental health disorders (depression and anxiety) be referred to other specialists (psychologist/psychotherapist) for psychological therapy [23], which is the case of approximately 20% of patients consulting a GP in different European countries [24, 25]. The most important obstacles to referral are the cost of psychotherapy because private psychologists are often not covered by the health insurance system, delay to have an appointment with a mental health specialist, patients’ reluctance to undergo psychotherapy and lack of cooperation between GPs and mental health medical centers [17–20].

In France, there are no systematic data regarding the management of work-related CMD in primary care. Therefore, although psychological support may facilitate return to work, we do not know how often psychological support is actually provided to persons who have work-related mental health disorders [26]. Occupational medicine services aim to reintegrate persons back at work, measure their disability level and prevent a worsening of the situation [27]. Overall, referral rates to occupational physicians are low (OP) [28] and there is little knowledge regarding collaboration between GPs and occupational medicine services [29].

The aims of this study are thus:

1. to describe the management of patients with work-related CMD in primary care practice. In particular, we estimate the prevalence of prescription of psychotropic medication, referral to a psychologist/psychiatrist, sick leave and referral to and OP;
2. to study whether the management of patients with work-related CMD varies with patient, work, contextual or GP characteristics.

## Methods

### Design and study population

The Heracles cross-sectional study was conducted from April to August 2014 among working individuals consulting a GP in the Nord - Pas-de-Calais region, now part of Hauts de France region [10]. Participating GPs were asked to include working patients (at least 6 months of full-time employment during the preceding 12 months) aged from 18 to 65 years they saw during the study period, whatever the reason of appointment. GPs were selected in a way that was proportional to their geographical distribution. The study was proposed to approximately a quarter of GPs in the Nord - Pas-de-Calais region (*n* = 1000).

To allow the recruitment of a representative sample of patients, at study initiation, a schedule was provided to each GP indicating the number of patients to include per time slot (16 possible slots). GPs gave each patient an information form, invited them to participate and asked them to sign an inform consent. More details about the recruitment process have previously been published [10].

This study was conducted by the Sentinelles network, part of the INSERM-Paris Sorbonne University research unit UMR-S 1136 [30].

### Data

After their regular appointment, GPs interviewed participating patients for the purposes of the study. Only patients diagnosed by the GP with a work-related CMD in the preceding 12 months or at the time of the appointment (depressive disorder, anxiety disorder or alcohol abuse) were included in the analyses. Collected data were:

Patient management in the preceding 6 months or at the time of the appointment (completed by the GP):

- Psychological support;
- Prescription of psychotropic medication (antidepressants, anxiolytics and hypnotics);
- Referral to a psychologist/psychiatrist;
- Sick leaves prescription;
- Referral to OP.

Patients’ characteristics:

- Sociodemographic (age, gender, family status, family income, level of education);
- Psychiatric and somatic health
- Suicidal risk identified by the MINI [31];
- Occupational grade [32], classified into three groups: blue (farmer/manual worker), pink (technician/associate professional/clerk/service worker) and white collar workers (manager/professional) [33];
- Company size;
- Job instability assessed based on the type of contract (temporary vs. permanent)
- Reason for medical appointment (somatic, psychological, chronic disease management).

GP’s characteristics

- Sociodemographic (age, gender);
- GP’s opportunity to collaborate with mental health specialists.

Work characteristics.

We used 20 questions evaluating psychosocial work characteristics, based on the international scientific literature and proposed by experts in the field [34]. These questions explored 6 dimensions of psychosocial work characteristics: work intensity (5 items), emotional demand (6 items), autonomy (2 items), social work relations (3 items), conflict of values (2 items) and job insecurity (2 items).

We also retrieved data concerning contextual characteristics [26, 27]: social deprivation (loneliness, single parenthood, widowhood/divorce) and material deprivation (unemployment, income, level of not graduated).

### Statistical analysis

First, we described the management of work-related CMDs.

Then we considered associations between different types of management of work-related CMDs and explanatory variables: patient-related, work-related, GP-related and contextual. The dependent variables were different types of management of work-related CMDs: prescription of psychotropic medication; sick leave; referral to an OP; referral to a psychologist/psychiatrist. We used modified Poisson regression with robust variance models [35]: first, in bivariate models all variables (*n* = 29) were tested; second, in multivariate models with a stepwise backward elimination procedure (all characteristics with *p* ≥ 0.2 in the bivariate analysis [*n* = 7 to 10 variables depending on the outcome] were included in multivariate models). Analyses were at first stratified by gender; we did not observe any differences between men and women, therefore gender was controlled for in our statistical analyses.

All analyses were performed using GNU R software version 3.1.1.

### Ethical approval

The study benefits from a standing authorization from the French independent administrative authority protecting privacy and personal data (CNIL), n°471,393 to conduct research among GPs and their patients.

## Results

### Sample description

121 GPs completed the study and included 2027 patients. 533 had a work-related CMD and were included in the statistical analyses. Patients were mostly female (57.8%) and almost half of them were aged between 36 to 50 years. 46.0% (*n* = 239) had depression, 75.2% (*n* = 391) anxiety and 8.8% (*n* = 46) an alcohol-related disorder. Patient, GP, work and contextual characteristics are described in Table 1.

**Table 1:** Description of study population. Héraclès study. Nord – Pas-de-Calais, France. 2014

	N	%
**Patient characteristics**		
Gender		
Male	225	42.2
Female	308	57.8
Age group		
[18–35]	123	23.1
[36–50]	249	46.8
[51–65]	160	30.1
Occupational category		
Blue collar	55	10.8
White collar	141	27.6
Pink collar	314	61.6
Educational level		
< High school degree	225	42.2
≥ High school degree	308	57.8
Family status		
Lives alone	151	28.4
Lives with a partner or parents	381	71.6
Family income		
[0–3000 euros]	154	34.7
3000 + euros	290	65.3
Number of workers in the company		
1 to 9	106	20.5
10 +	410	79.5
Psychiatric history *(yes* vs *no)*	95	18.3
Somatic history *(yes* vs *no)*	136	26.1
Purpose of appointment with GP *(yes* vs *no)*		
Somatic	249	46.7
Psychological	296	55.5
Chronic disease management	81	15.2
Common mental disorder *(yes* vs *no)*		
Depression	239	46.0
Anxiety	391	75.2
Alcohol	46	8.8
Suicidal risk (MINI questionnaire)	138	25.9
Past unemployment *(yes* vs *no)*	157	29.5
Job instability *(yes* vs *no)*	198	38.4
**GPs characteristics**		
GP’s gender		
Male	332	62.3
Female	201	37.7
GP’s age		
[18–49]	194	36.4
50 +	339	63.6
Opportunity to work with mental health specialists		
High	291	56.4
Low	225	43.6
**Contextual characteristics**		
Social deprivation		
High	159	29.8
Low	374	70.2
Material deprivation		
High	266	49.9
Low	267	50.1
**Work characteristics**		
Work intensity		
High	202	37.9
Low	331	62.1
Emotional demands		
High	222	41.7
Low	311	58.3
Autonomy		
High	100	18.8
Low	433	81.2
Conflict of values		
High	288	54.0
Low	245	46.0
Social relationships at work		
High	67	12.6
Low	466	87.4
Job Insecurity		
High	209	39.2
Low	324	60.8

### Description of GPs’ management of work-related CMDs (Table 2)

Among the 533 patients with work-related CMDs, 88.0% (*n* = 469) received psychological support from their GP and 82.4% (*n* = 439) a prescription for a psychotropic medication. The most prescribed psychotropic medications were anxiolytics (62.1%), antidepressants (41.8%) and hypnotics (23.1%).

**Table 2:** Description of GPs’ management of work related common mental disorders. Héraclès study. Nord – Pas-de-Calais, France. 2014

	N	%
Psychological support *(yes* vs *no)*	469	88.0
Prescription of psychotropic medication *(yes* vs *no)*	439	82.4
Antidepressants	223	41.8
Anxiolytics	331	62.1
Hypnotics	123	23.1
Sick leave prescription related to mental disorders *(yes* vs *no)*	270	50.7
Average length of sick leave *(in weeks)*	5.36	
Referral		
To occupational physician	139	26.1
To psychologist/psychiatrist	212	39.8
To psychiatric emergency	16	3.0

39.8% of patients were referred to a psychologist or psychiatrist. Sick leave was prescribed to 50.7% (*n* = 270) of patients, with an average length of 5.36 weeks.

26.1% of patients were referred to an occupational physician.

### Factors associated with GPs’ management of work-related CMDs

#### Prescription of psychotropic medications

We observed a higher rate of prescription of psychotropic medication for patients with depressive disorders (RR = 1.08 [1.01–1.16]) and patients who saw their physician for psychological purposes (RR = 1.25 [1.14–1.36]). We observed a higher level of prescription of psychotropic medication for older participants (51 to 65 years) compared to those who were 18 to 35 years old (RR = 1.14 [1.03–1.27]) (Table 3). Prescription of psychotropic medication was not associated with any psychosocial work factors.

**Table 3:** Factors associated with the prescription of psychotropic medication, Héraclès study, France, 2014. Poisson regression models (*n* = 518)

	RR*	CI 95%	p
Age group			
[18–35]	1.00	–	0.56
[36–50]	1.03	[0.93–1.14]	
[51–65]	1.14	[1.03–1.27]	
Psychological purpose of consultation	1.25	[1.14–1.36]	< 0.01
Depression detected by GP	1.08	[1.01–1.16]	0.04

* adjusted on gender

#### Referral to a psychologist or psychiatrist

There was a higher rate of referral to a psychologist or psychiatrist for patients with depressive disorders compared to patients with other CMDs (RR = 1.71 [1.34–2.18]), patients who saw their GP for psychological purposes (RR = 1.32 [1.06–1.65]) and those with a suicidal risk (RR = 1.49 [1.22–1.82]). Patients with a somatic or psychiatric history were more likely to be referred to a psychologist or psychiatrist with RRs of respectively 1.24 and 1.26. There was a higher rate of referral for patients whose GP had more opportunity to collaborate with mental health specialists (RR = 1.32 [1.05–1.66]). Patients living in an area with high material deprivation were more likely to be referred to a psychologist or psychiatrist (RR = 1.32 [1.08–1.62]) (Table 4). Referral to a psychologist or psychiatrist was not associated with any psychosocial work factors.

**Table 4:** Factors associated with referral to a psychologist or psychiatrist. Héraclès study. Nord – Pas-de-Calais, France. 2014. Poisson regression models (*n* = 475)

	RR*	CI 95%	p
Depression detected by GP	1.71	[1.34–2.18]	< 0.01
Suicidal risk	1.49	[1.22–1.82]	< 0.01
Psychological purpose of consultation	1.32	[1.06–1.65]	0.01
Psychiatric history	1.26	[1.01–1.57]	0.04
Somatic history	1.24	[1.02–1.52]	0.03
Opportunity to work with mental health specialists	1.32	[1.05–1.66]	0.02
Material deprivation	1.32	[1.08–1.62]	< 0.01

* adjusted on age and gender

#### Sick leave

Patients with depressive disorder had a higher rate of sick leave (RR = 1.18 [1.01–1.39]) compared to patients with other work-related CMDs as did patients who saw their physician for psychological reasons (RR = 1.83 [1.49–2.24]). For patients working in a big company the relative risk (RR) of sick leave was higher compared to patients working in smaller companies. Psychosocial work factors such as high emotional demands and high social relationships at work were significantly associated with sick leave with RRs respectively of 1.33 and 0.65 (Table 5).

**Table 5:** Factors associated with sick leave. Héraclès study. Nord – Pas-de-Calais, France. 2014. Poisson regression models (*n* = 502)

	RR*	CI 95%	p
Depression detected by GP	1.18	[1.01–1.39]	0.05
Psychological purpose of consultation	1.83	[1.49–2.24]	< 0.01
Number of workers in the company			
1 to 9	1.00	–	0.04
10 +	1.26	[1.01–1.58]	
Emotional demands at work *(high* vs *low)*	1.33	[1.13–1.56]	< 0.01
Social relationships at work *(high* vs *low)*	0.65	[0.46–0.92]	0.01

* adjusted on age and gender

#### Referral to an occupational physician

The rate of referral to an OP was higher for patients with depressive disorder compared to patients with other work-related CMDs (RR = 1.34 [1.00–1.79]). White collar workers had a lower rate of referral compared to blue collar workers (RR = 0.51 [0.31–0.88]). High work intensity was significantly associated with OP referral (RR = 1.41 [1.06–1.88]) (Table 6).

**Table 6:** Factors associated with referral to an occupational physician. Héraclès study. Nord – Pas-de-Calais, France. 2014. Poisson regression models (*n* = 485)

	RR*	CI 95%	p
Occupational category			
Blue collar	1.00	–	0.03
White collar	0.51	[0.31–0.88]	
Pink collar	0.91	[0.58–1.42]	
Depression detected by GP	1.34	[1.00–1.79]	0.05
Work intensity *(high* vs *low)*	1.41	[1.06–1.88]	0.02

* adjusted on age and gender

## Discussion

### Summary

Our study reports the management of patients with work-related CMD in primary care. GPs provided psychological support to nearly 90% of patients; they prescribed psychotropic medication to more than 80%. They referred approximately 40% of patients to a psychologist or psychiatrist. Sick leave was prescribed to more than half of the patients, with an average length of 5.36 weeks. 26.1% of patients were referred to an OP. Low referral rates to psychologists, psychiatrists and OPs indicate that GPs often manage these patients alone. This could be the GPs choice if he or she has sufficient skills in this area, or a sign of lack of cooperation with mental health and occupational health specialists.

No work-related factors were associated with neither psychotropic medication prescription nor referral to a psychologist or psychiatrist. Work-related factors were associated with sick leave prescription and referral to an OP. Higher rates of sick leave were related with suicidal risk, employment in a large company, as well as emotional demands and social relationships at work. Referral to an OP was more frequent for patients with depressive disorders and high work intensity, while it was lower for white collar workers.

### Comparison with existing literature

#### GP’s management of patients with work-related CMD

Our results are consistent with previous studies on GP’s management of patients with CMD, indicating that there is little special management in case of work-related CMD, compared to CMDs outside the context of work. In France, on average, more than 90% of the GPs prescribed psychotropic medication to patients with depressive disorders [36]. Another study found that 80% of patients receiving antidepressants or tranquilizers had a depressive or anxiety disorder diagnosed by their GP [19].

Rates of psychological support observed in our study in case of work-related CMD are also consistent with the scientific literature in CMD. In France, as in other countries, 2/3 of GPs offer their patients psychological support, which is often associated with the prescription of psychotropic medication [16–18, 37]. Psychological support is particularly important in case of work-related issues, as it helps in the process of returning to work [26].

Referral rates to psychiatrists or psychologists in case of work-related CMD are also consistent with previous studies [25, 28]. Low rates of referral could be related to difficulties in accessing these specialists, lack of coverage by the national health insurance system, patients’ refusal or lack of the GPs lack of training [18, 36]. This could also be the GP’s choice [15].

Two results should be highlighted in the specific context of work-related CMDs.

First, our study reports a low rate of referral to OPs that could be explained by suspicion about the physician’s lack of impartiality. OPs are often employed by companies and could be perceived as subject to their employer. Additionally, GPs may not know the role of OPs in detail [28, 38].

Second, rates of sick leave in case of work-related CMD are consistent with a Norwegian study where 45% of patients with CMD have a sick leave prescribed by the GP [39]. The average length of sick leave is compatible with data from the French national health insurance, showing that there is a high rate of long term sick leave for individuals with CMD [40].

#### Factors associated with GP’s management of patients with work-related CMD

Our study highlights several factors associated with the management of patients with work-related CMDs. In the literature, very few studies explored factors associated with GPs’ management of CMDs and almost none for work-related issues and referral to OP by GP.

The relationship between the prescription of psychotropic drugs and the patient’s age in case of depression [41] or psychologic complaints and more severe mental disorders [42] has previously been described.

Referral to a mental health specialist has been associated with psychiatric history, depressive symptoms, suicidal risk [43, 44] and the opportunity to work with mental health specialists [45].

For sick leave, our results indicate that GPs follow trends that have been highlighted among workers. Depressive disorders are the first cause of sickness certification for CMDs [46]. It is interesting to note that GPs take into account dimensions of work to prescribe sick leave: they are more frequent in case of low social support at work and high psychological demands [47, 48]. The size of the company is also important, indicating that it is easier to be absent from work when there are colleagues who can replace you. This has been highlighted in a Japanese study [49].

Our study explores for the first time referral to OPs by GPs. We show that this is associated with work-characteristics. Fewer referrals to OPs for white collar have to be explored further. They may be related to mistrust of the relationship between OPs and the company, especially for managers. GP contact the OP more frequently in case of higher work intensity, which makes sense when trying to adapt working conditions.

### Strengths and limitations

As described in previous articles based on the Héraclès study, some limitations have to be acknowledged [10, 50, 51]. First, there is possible selection bias of GPs. Participating GPs could be especially interested in CMDs because of personal interest or patients’ rate of CMDs. Therefore, participating GPs could have a better experience of managing those patients and thus a different practice towards them. However, physicians who participated were representative of the Nord – Pas-de-Calais region GPs, thus limiting this bias. Second, our study was conducted in the Nord - Pas-de-Calais, a region with a low density of medical health specialist [52]. This could influence GPs’ practice, especially for referral to other health professionals. Moreover, low income in this area could led to a more frequent management of CMDs by the GP. Another possible limitation is the absence of standard procedures for GPs to diagnose CMD. In France such procedures do not exist in primary care. Finally the definition of the link between work and CMDs could be a limiting factor, even if we relied on the WHO definition of work-related disorders and the scientific literature [1, 53–55]. Indeed, there is no validated and consensual definition of work-relatedness of psychological difficulties.

Despite these limitations, the results of this study are valuable, because to our knowledge, this is the first study in Europe to study the management of patients with work-related CMDs by the GP. Moreover, contrary to studies in occupation settings, our study was conducted among primary care patients, which include a large panel of workers in the labour force including workers who have a poor relation with occupational practice (independent workers, workers in small companies or workers who do not have an OP, etc.). An international study shows that the average occupational health services coverage of workers is 24.8% [55]. Finally, study questionnaires were completed by the GP who is often the referring physician and is well informed of the patient’s medical history and can assess if the disorder is related to work.

## Conclusion

Our study is one of the first to study the management of work-related CMDs by GPs. Our results emphasize the major role of GPs in the management of those patients (psychological support, psychotropic use and sick leaves), with weak referrals to other mental health specialists or OPs. There is little special management in case of work-related CMDs, compared to CMDs without the context of work, except for sick leave and referral to an OP. Factors associated with the different types of management could help to better understand the way GPs manage those patients and could be a starting point to implement collaborative care, especially with OP.

## Data Availability

The datasets generated and/or analysed during the current study are not publicly available but are available from the corresponding author on reasonable request.

## Abbreviations

CMD: Common mental disorders
GP: General practitioner
OP: Occupational physician
RR: Relative risk
